# Essential oil–based interventions for chemotherapy-induced nausea and vomiting in women with breast cancer: a systematic review and meta-analysis

**DOI:** 10.3389/fmed.2026.1897656

**Published:** 2026-07-23

**Authors:** Kaikai Jin, Shengli Yan, Leilai Xu

**Affiliations:** 1 Department of Breast Surgery, The First Affiliated Hospital of Zhejiang Chinese Medical University (Zhejiang Provincial Hospital of Chinese Medicine), Hangzhou, China; 2 Department of General Surgery, The Affiliated Traditional Chinese Medicine Hospital of Shihezi University, Shihezi, China

**Keywords:** aromatherapy, breast cancer, chemotherapy-induced nausea and vomiting, essential oils, meta-analysis, supportive care

## Abstract

**Objectives:**

Chemotherapy-induced nausea and vomiting (CINV) remains common in patients with breast cancer despite standard antiemetic therapy. Essential oil–based interventions, including inhalation aromatherapy and essential oil–derived preparations, are increasingly used as supportive care, but their efficacy remains uncertain.

**Methods:**

We conducted a systematic review and meta-analysis of RCTs comparing essential oil–based interventions with control conditions in adults with breast cancer receiving chemotherapy. PubMed, Embase, Cochrane CENTRAL, and Web of Science were searched through 14 February 2026. The acute phase was defined as within 24 h after chemotherapy. Continuous outcomes were pooled as Hedges’ g standardized mean differences (SMDs) with 95% CIs using random-effects REML models. RoB 2, GRADE, and Knapp–Hartung sensitivity analyses were applied.

**Results:**

Four RCTs were included. For acute nausea severity (4 RCTs, *n* = 336), essential oil–based interventions were associated with reduced symptoms versus control (SMD = −0.49, 95% CI −0.76 to −0.22; I^2^ = 35.42%). For vomiting frequency (2 RCTs, *n* = 144), the pooled effect was not significant (SMD = −0.25, 95% CI −0.75 to 0.25; I^2^ = 56.52%). For vomiting severity/score (2 RCTs, *n* = 192), a modest reduction was observed in the primary REML analysis (SMD = −0.31, 95% CI −0.59 to −0.03; I^2^ = 0%), but not after Knapp–Hartung adjustment. RoB 2 identified one study at low risk, two with some concerns, and one at high risk of bias. GRADE certainty was low for acute nausea and very low for vomiting outcomes.

**Conclusion:**

Essential oil–based interventions may reduce acute nausea in women with breast cancer receiving chemotherapy, but evidence remains limited, heterogeneous, and of low to very low certainty. Findings should be interpreted cautiously because the evidence included inhalation aromatherapy trials and one exploratory oral peppermint extract trial. These interventions should not replace guideline-based antiemetic therapy. Larger, well-designed RCTs with prospectively registered protocols are needed.

**Systematic review registration:**

https://www.crd.york.ac.uk/prospero/, identifier [CRD420261308323].

## 1 Introduction

Breast cancer remains the most commonly diagnosed malignancy among women worldwide, and chemotherapy continues to be a cornerstone of treatment across multiple disease stages (1). However, chemotherapy-induced nausea and vomiting (CINV) remains one of the most distressing adverse effects of treatment and may substantially impair quality of life, nutritional intake, treatment adherence, and overall chemotherapy tolerance (2). Although guideline-based antiemetic regimens have markedly improved emesis control, nausea—particularly in the acute phase—remains insufficiently controlled in many patients (3).

In recent years, aromatherapy and essential oil–based interventions have been increasingly explored as integrative supportive care strategies in oncology (4). These interventions are generally non-invasive, low-cost, and easy to administer, and they may provide additional symptom relief when used alongside standard antiemetic therapy (5). Nevertheless, the available clinical evidence remains inconsistent. Some studies have reported improvements in chemotherapy-related nausea, whereas findings for vomiting outcomes have been less consistent, likely due to differences in essential oil type, delivery route, outcome measures, and timing of assessment (6).

Previous reviews have examined aromatherapy in patients with cancer, but many pooled analyses included mixed cancer populations, heterogeneous interventions, and non-uniform outcome definitions, which limited the interpretability of findings for specific clinical settings (5, 7, 8). In particular, evidence focused on patients with breast cancer receiving chemotherapy and on the acute phase after chemotherapy remains limited. In addition, nausea and vomiting are often analyzed together despite important differences in symptom mechanisms and clinical measurement.

Therefore, we conducted a systematic review and meta-analysis of randomized controlled trials to evaluate the effects of essential oil–based interventions on chemotherapy-induced nausea and vomiting in patients with breast cancer receiving chemotherapy, with particular focus on the acute phase after chemotherapy.

## 2 Methods

### 2.1 Information sources and search strategies

This systematic review and meta-analysis was registered in PROSPERO (CRD420261308323) and conducted in accordance with the Preferred Reporting Items for Systematic Reviews and Meta-Analyses (PRISMA) statement (9). We searched PubMed, Embase, the Cochrane Central Register of Controlled Trials (CENTRAL), and Web of Science from inception to 14 February 2026. Search strategies combined controlled vocabulary, where applicable, and free-text keywords covering three concept blocks: breast cancer, chemotherapy-induced nausea and vomiting (CINV), and aromatherapy/essential oils. Strategies were adapted to each database’s indexing and syntax. Searches were limited to English-language publications.

The reference lists of included studies and relevant reviews were also screened for additional eligible records, but no additional records were identified. The complete electronic search strategies for all databases, provided in Supplementary Material 1, were used to generate the counts for the PRISMA flow diagram.

### 2.2 Eligibility criteria

Studies were included if they met all of the following criteria:

(1) Participants: adults (≥18 years) with a confirmed diagnosis of breast cancer receiving chemotherapy;
(2) Interventions: essential oil–based interventions, defined operationally as interventions involving essential oils (volatile oils) or essential oil–derived preparations, administered via any route (e.g., inhalation, topical application/massage, or oral essential oil–related preparations), used alone or as an adjunct to routine care;
(3) Comparators: studies with a control group receiving routine care/standard care, placebo, no intervention, or other non-essential-oil control conditions;
(4) Outcomes: nausea, vomiting, or both reported as study outcomes;
(5) Study design: randomized controlled trials (RCTs).

For meta-analysis, the acute phase was prespecified as outcomes assessed within 24 h after chemotherapy. If a study reported multiple assessments within the first 24 h, the earliest post-chemotherapy measurement within this window was selected to ensure comparability across trials.

Studies were excluded if they: (1) did not include a control group; (2) were not randomized controlled trials, including non-randomized, quasi-randomized, observational, review, or conference abstract-only reports without sufficient extractable data; (3) evaluated essential oil–based interventions combined with other interventions such that the independent effect of the essential oil–based component could not be isolated; or (4) lacked key quantitative data required for meta-analysis (e.g., group sample size and dispersion measures necessary to compute effect sizes).

### 2.3 Protocol deviations/amendments

The original PROSPERO registration was focused on aromatherapy interventions for chemotherapy-induced nausea and vomiting in women with breast cancer. The electronic search strategy included aromatherapy and essential oil terms, but the original eligibility scope and initial study selection were conducted according to an aromatherapy-focused protocol. During manuscript revision, and in response to peer-review comments regarding the inclusion of one trial using oral peppermint extract, we revised the operational eligibility definition from “aromatherapy” to “essential oil–based interventions.” The revised definition included interventions involving essential oils or essential oil–derived preparations administered via any route, including inhalation aromatherapy and oral essential oil–related preparations.

This change was made after the initial screening process and after the oral peppermint extract trial had been identified and its design and outcome data had been examined during manuscript preparation and revision; therefore, it is reported here as a protocol deviation/amendment rather than as a prospectively specified amendment. It was intended to transparently reflect the intervention characteristics of the included oral peppermint extract trial rather than to imply clinical equivalence between oral peppermint extract and inhalation aromatherapy. The original PROSPERO record had not been prospectively amended before screening or data extraction. An update to the PROSPERO record is being prepared to document this protocol amendment.

As a corrective action, two reviewers repeated the title/abstract screening of all 161 deduplicated records and the full-text eligibility assessment of all 11 reports retrieved for full-text review according to the broadened essential oil–based eligibility criteria. Discrepancies were resolved through discussion and consensus. No additional eligible studies were identified, and the final included study set remained unchanged. Therefore, the oral peppermint extract trial is treated as part of an exploratory broadened-scope analysis, and this protocol deviation/amendment is considered when interpreting the applicability and certainty of the findings.

### 2.4 Study selection and data extraction

All records were imported into reference management software and deduplicated prior to screening. Two reviewers independently screened titles and abstracts, followed by full-text assessment of potentially eligible reports. Discrepancies were resolved through discussion and consensus.

Data were extracted independently by two reviewers using a standardized form. Extracted items included: study identifiers (author/year, country/setting), design characteristics (parallel/crossover, blinding), participant characteristics (sample size, eligibility criteria, age), chemotherapy context, intervention details (essential oil type, delivery mode, dose/intensity where reported, frequency, duration, and adherence/implementation), comparator details, outcome definitions and timepoints, and numerical outcome data required for synthesis (group sample size, means, and standard deviations). When multiple acute timepoints were reported, the prespecified acute timepoint (as described above) was used for meta-analysis.

### 2.5 Risk of bias assessment

Risk of bias was assessed using the Cochrane Risk of Bias 2 (RoB 2) tool (10) across five domains: (1) bias arising from the randomization process, (2) deviations from intended interventions, (3) missing outcome data, (4) measurement of the outcome, and (5) selection of the reported result. Each domain was judged as “low risk,” “some concerns,” or “high risk,” leading to an overall risk-of-bias judgment.

Risk of bias was assessed with attention to the outcomes included in the synthesis, including acute nausea severity, vomiting frequency, and vomiting severity/score. Because nausea and vomiting outcomes were patient-reported or symptom-based, we explicitly considered whether blinding could have been compromised by recognizable odor, taste, or administration route. This consideration was particularly relevant for trials using non-odor-matched inhalation comparators or oral peppermint extract. Where blinding procedures, allocation concealment, or outcome reporting were insufficiently described, judgments for deviations from intended interventions and measurement of outcomes were made conservatively. Two reviewers assessed risk of bias independently and resolved disagreements through discussion and consensus.

### 2.6 Synthesis and statistical analysis

All quantitative analyses were performed using Stata/MP 16.0 (StataCorp LLC, College Station, TX, United States). Continuous outcomes were synthesized using the standardized mean difference (SMD; Hedges’ g) with 95% confidence intervals (CIs). Because higher scores/frequencies indicated worse symptoms, negative SMD values indicated improvement with essential oil–based interventions. Vomiting frequency was analyzed using SMD because the included trials reported vomiting episodes as aggregate continuous summary data using group means and standard deviations, and individual participant-level count data or rate data were unavailable. Therefore, count-based models could not be applied. This approach allowed synthesis using the available aggregate data but reduced the direct clinical interpretability of the vomiting frequency estimate.

Meta-analyses were conducted using random-effects models to account for between-study variability, with between-study variance (τ^2^) estimated by restricted maximum likelihood (REML) (11). Statistical heterogeneity was assessed using Cochran’s Q and quantified using I^2^, interpreted as 0%–40% (might not be important), 30%–60% (may represent moderate heterogeneity), 50%–90% (may represent substantial heterogeneity), and 75%–100% (considerable heterogeneity) (12). Where heterogeneity was observed, potential clinical sources were explored by comparing study design, participant characteristics, intervention features (e.g., essential oil type and delivery method), comparator conditions, and outcome measurement tools/timepoints. Subgroup analyses and meta-regression were not performed because of the limited number of included studies for each outcome.

Robustness of pooled estimates was evaluated using the Knapp–Hartung (13) adjustment for random-effects meta-analysis. Because one included trial used oral peppermint extract rather than inhalation aromatherapy, we conducted a *post hoc* sensitivity analysis for the primary outcome of acute nausea severity by excluding this trial. Additional influence analyses were not performed because of the limited number of included studies. Publication bias and small-study effects were planned to be assessed using funnel plots and Egger’s regression test (14) when an outcome was informed by a sufficient number of studies; however, formal assessment was not performed because fewer than 10 studies were available for each outcome (15). All statistical tests were two-sided, and (*P* < 0.05) was considered statistically significant.

### 2.7 Assessment of certainty of evidence

The certainty of evidence for each outcome was assessed using the GRADE approach (16), considering risk of bias, inconsistency, indirectness, imprecision, and publication bias. Evidence from randomized trials started at high certainty and was downgraded when concerns were identified. A Summary of Findings (SoF) table (17) was prepared to present pooled effects and certainty ratings.

## 3 Results

### 3.1 Study selection

**FIGURE 1:**
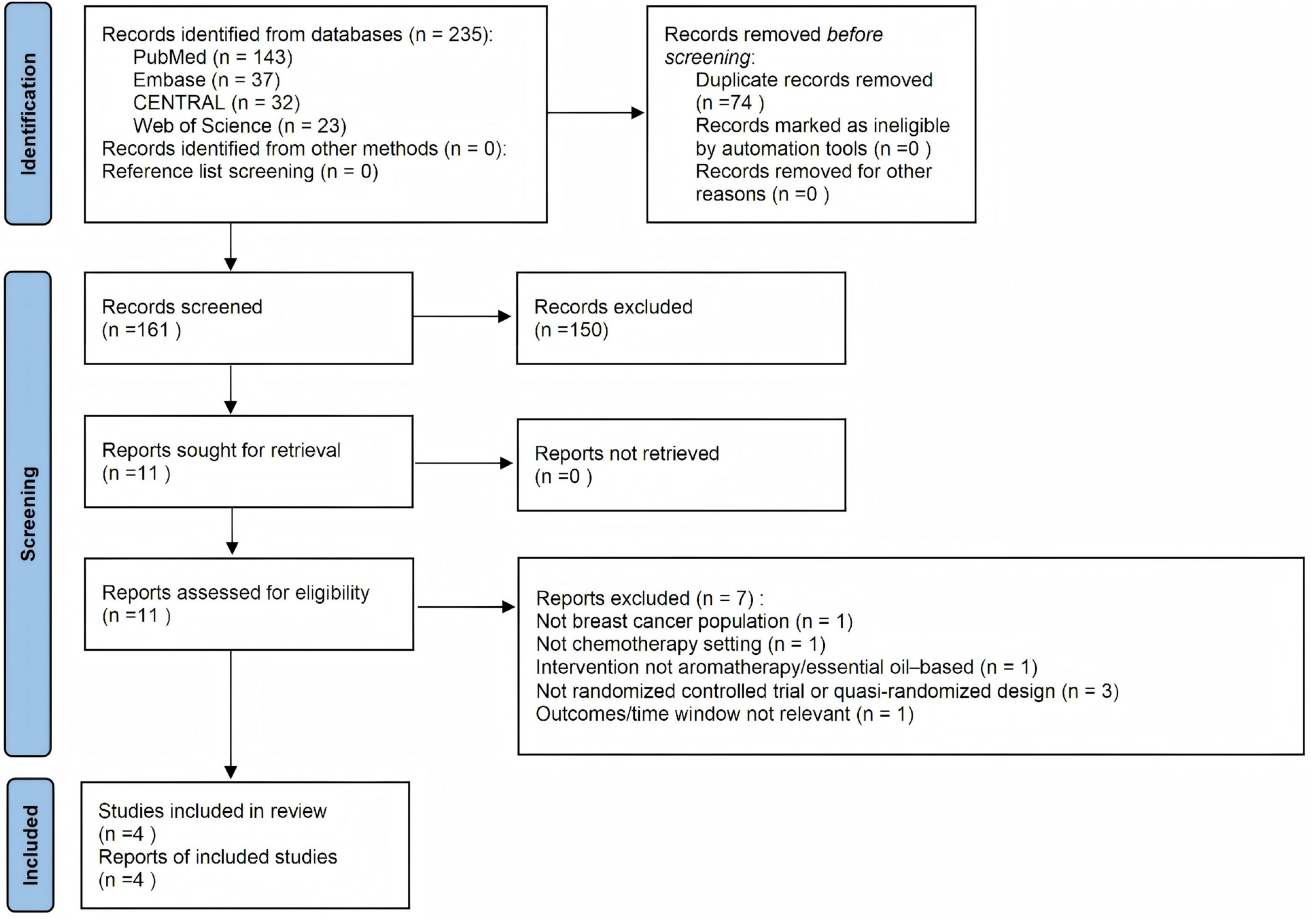
Preferred Reporting Items for Systematic Reviews and Meta-Analyses (PRISMA) 2020 flow diagram of study selection.

A total of 235 records were identified through database searching, including PubMed (*n* = 143), Embase (*n* = 37), the Cochrane Central Register of Controlled Trials (CENTRAL; *n* = 32), and Web of Science (*n* = 23). No additional records were identified through reference list screening. After removal of 74 duplicate records, 161 records remained for title and abstract screening. Of these, 150 records were excluded. The full texts of 11 reports were sought and assessed for eligibility, and seven reports were excluded for the following reasons: not breast cancer population (*n* = 1), not chemotherapy setting (*n* = 1), intervention not essential oil–based (*n* = 1), not randomized controlled trial or quasi-randomized design (*n* = 3), and outcomes/time window not relevant (*n* = 1). Ultimately, four studies were included in the qualitative synthesis, and all four were included in the meta-analysis (Figure 1). After the protocol deviation/amendment was identified, two reviewers repeated title/abstract screening of all 161 deduplicated records and full-text eligibility assessment of all 11 reports according to the broadened essential oil–based eligibility criteria; no additional eligible studies were identified.

### 3.2 Study characteristics

Four RCTs were included (Table 1), conducted in Iran (*n* = 3) and Malaysia (*n* = 1). Three trials used a parallel-group design (18–20), while Lua et al. (21) was a randomized crossover trial; to avoid potential carryover, only first-period data (30/30) were used for meta-analysis. Blinding was triple-blind in Jafarimanesh et al. (19), double-blind in Parsa et al. (20), single-blind in Lua et al. (21), and not reported in Eghbali et al. (18). Across studies, routine antiemetic treatment was provided in both intervention and control groups. However, the original reports did not provide sufficient detail to determine whether all antiemetic regimens fully aligned with current guideline-based recommendations. Chemotherapy regimens included AC, AC-T, mixed regimens, or HEC commonly used in breast cancer.

**TABLE 1:** Characteristics of included randomized controlled trials.

References	Country	Design (blinding)	Participants (I/C randomized; analyzed)	Chemotherapy/antiemetics	Intervention (oil/form; dose; route; frequency; duration)	Control (placebo/usual care)	Outcomes and window (acute time point for meta)
Eghbali et al., (18)	Iran	Parallel RCT; blinding not reported	Randomized 50/50; analyzed 50/50	AC; standard antiemetics	Peppermint EO, two drops on collar tissue; inhale 20 min, three times/day, for one night	Normal saline on tissue (placebo; non-odor-matched)	Rhodes index; 0–24 h post-chemotherapy (acute for meta: 0–24 h)
Jafarimanesh et al., (19)	Iran	Triple-blind RCT	Randomized 42/42; analyzed 42/42	Mixed regimens; routine antiemetics (both groups)	Oral peppermint extract (peppermint-based preparation), 40 drops/20 mL water; once daily for 7 days	Distilled water in 20 mL tap water (placebo)	VAS nausea/anorexia; vomiting frequency; 24 h post-chemotherapy (acute for meta: 24 h)
Lua et al., (21)	Malaysia	Randomized crossover; single-blind	Randomized 75; completed 60; analyzed for meta (period 1) 30/30	HEC common (FEC/TAC); standard antiemetics (both periods)	Ginger EO, two drops on necklace; passive inhalation; for 5 days	Ginger fragrance oil on necklace (odor-matched placebo)	VAS nausea (Day 1–5); vomiting frequency (acute for meta: Day 1)
Parsa et al., (20)	Iran	Parallel RCT; double-blind	Randomized 50/50; analyzed 46/46	AC-T; identical antiemetic protocol	Citrus aurantium EO, two drops to philtrum; inhale 20 min, three times/day; for 5 days	Sunflower oil (odorless placebo)	Rhodes index (Days 1–5); acute for meta: Day 1

AC, anthracycline + cyclophosphamide; AC-T, AC followed by taxane; EO, essential oil; HEC, highly emetogenic chemotherapy; RCT, randomized controlled trial. Acute phase was defined as within 24 h after chemotherapy; the acute time point was extracted as reported by each trial (0–24 h/24 h/Day 1). Lua et al. (21) was a crossover trial; first-period data were extracted for meta-analysis (30/30). Jafarimanesh et al. (19) administered oral peppermint extract and was categorized as a peppermint-based essential oil–related intervention. Higher scores/frequencies indicate worse symptoms for the extracted outcomes; negative standardized mean difference (SMD) values favor essential oil–based interventions.

Essential oil–based interventions varied in type and delivery: peppermint [inhaled peppermint EO in Eghbali et al. (18); oral peppermint extract in Jafarimanesh et al. (19)], ginger EO inhalation via necklace [Lua et al. (21)], and Citrus aurantium EO inhalation [Parsa et al. (20)]. Control conditions were placebo comparators matched by procedure (normal saline on tissue; distilled water; odor-matched fragrance oil; sunflower oil). Outcomes were assessed using the Rhodes Index [Eghbali et al. (18), Parsa et al. (20)] or VAS-based ratings [Lua et al. (21), Jafarimanesh et al. (19)], with vomiting frequency additionally recorded in Lua et al. (21), Jafarimanesh et al. (19). The acute time point used for meta-analysis followed the prespecified window (≤ 24 h): 0–24 h [Eghbali et al. (18)], 24 h [Jafarimanesh et al. (19)], and Day 1 [Lua et al. (21), Parsa et al. (20)].

### 3.3 Risk of bias

**FIGURE 2:**
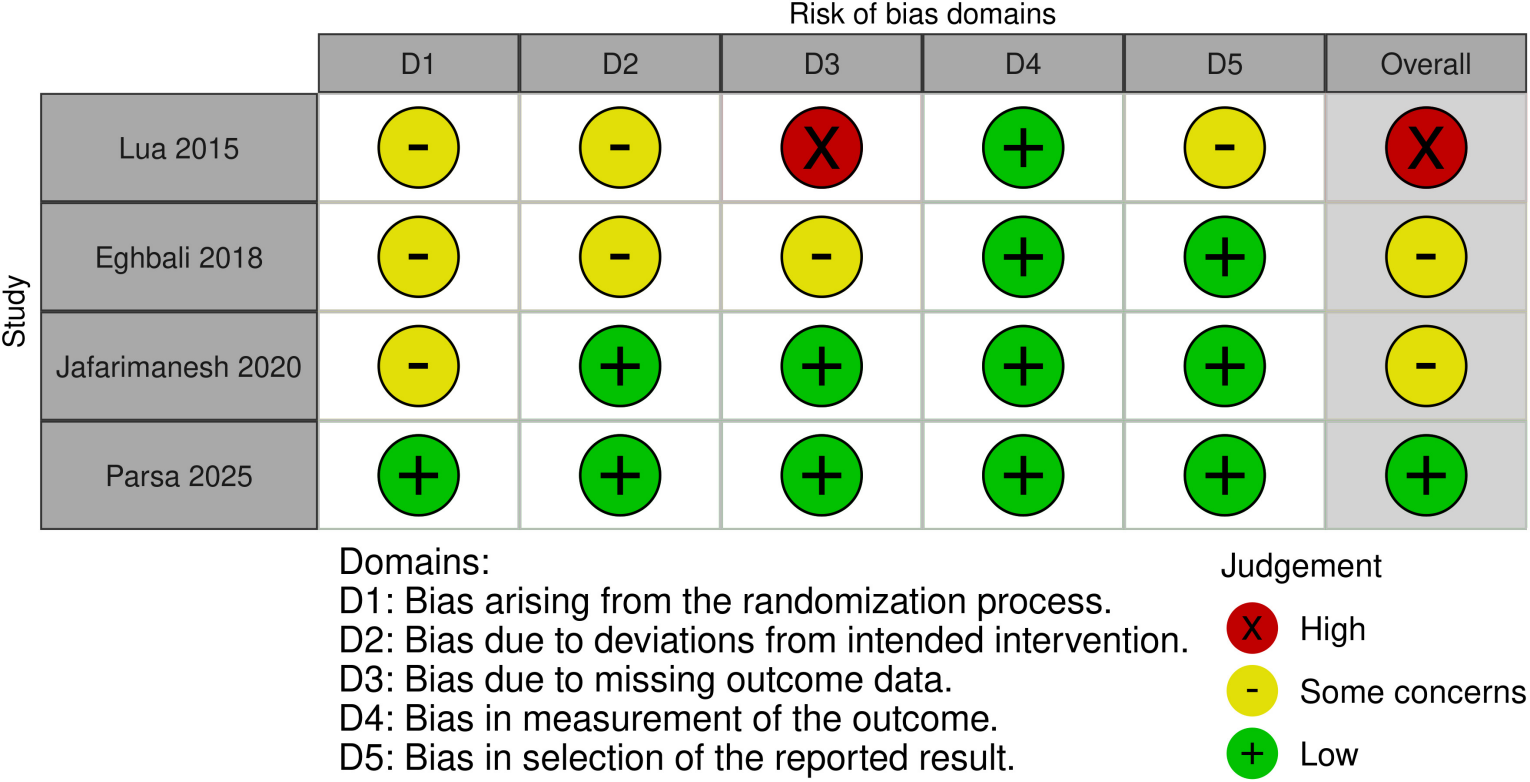
Risk-of-bias assessment of included trials using the Cochrane RoB 2 tool. Traffic-light plot showing domain-level and overall risk-of-bias judgments for each included randomized controlled trial. D1, bias arising from the randomization process; D2, bias due to deviations from intended interventions; D3, bias due to missing outcome data; D4, bias in measurement of the outcome; D5, bias in selection of the reported result.

Results of the RoB 2 assessment for the included RCTs are presented in Figure 2, and the domain-level distribution is summarized in Supplementary Figure 1. Risk of bias was considered for the synthesized outcomes, including acute nausea severity, vomiting frequency, and vomiting severity/score where data were available. Outcome-specific judgments were generally similar within each trial because the same trial conduct, blinding procedures, and symptom reporting mechanisms applied to the nausea and vomiting outcomes. However, because these outcomes were subjective or symptom-based, possible recognition of the intervention by odor, taste, or administration route was considered when judging bias due to deviations from intended interventions and bias in measurement of the outcome.

Overall, one trial was judged as low risk of bias, two trials were judged as having some concerns, and one trial was judged as high risk of bias for the primary nausea outcome, with broadly similar concerns for vomiting-related outcomes where data were available. The most common concerns were related to insufficient methodological detail regarding randomization, allocation concealment, and blinding. Possible compromised blinding was particularly relevant for non-odor-matched inhalation comparisons and for the oral peppermint extract trial. D3 contained the only high-risk domain judgment because of missing outcome data. These risk-of-bias concerns were incorporated into the interpretation of the certainty of evidence.

### 3.4 Acute nausea severity

**FIGURE 3:**
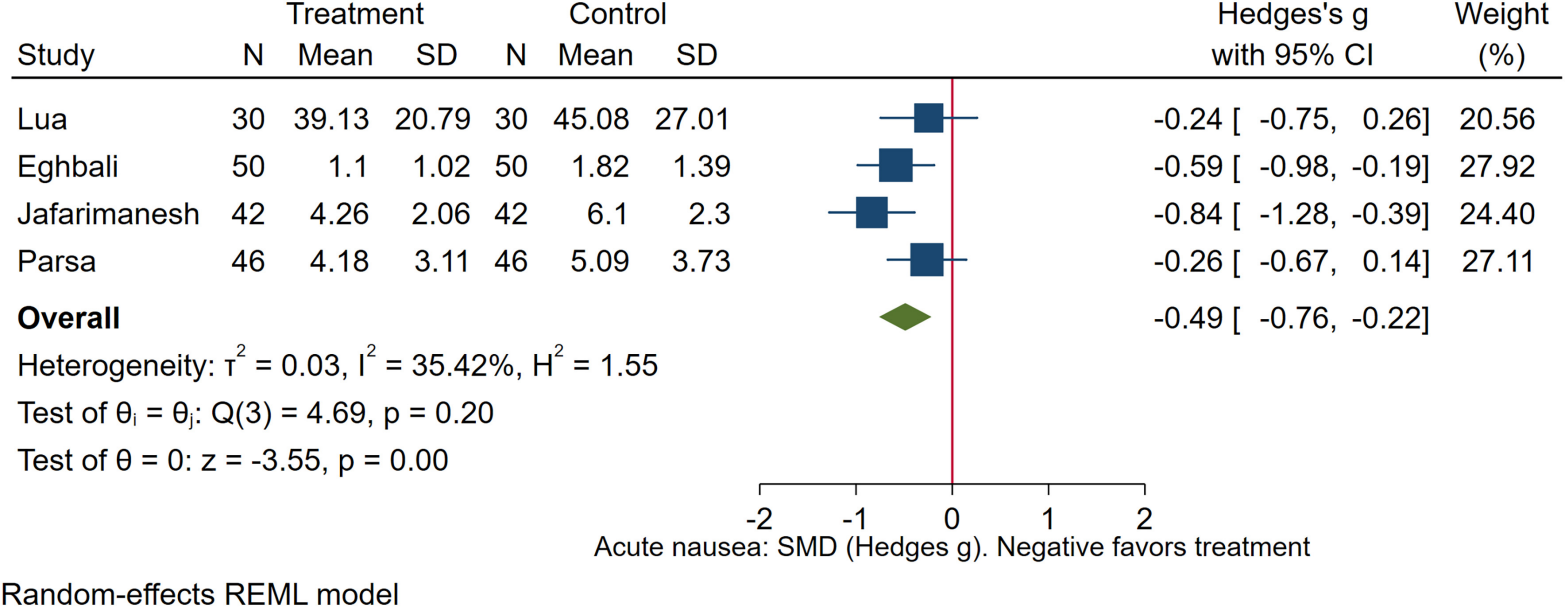
Forest plot of acute nausea severity comparing essential oil–based interventions versus control. Effect sizes are presented as standardized mean differences [standardized mean differences (SMDs), Hedges’ g] with 95% confidence intervals using a random-effects restricted maximum likelihood (REML) model. Negative values favor essential oil–based interventions.

Four RCTs (*n* = 336) were included in the meta-analysis of acute nausea severity (Figure 3). Compared with control, essential oil–based interventions were associated with a significant reduction in acute nausea severity [SMD (Hedges’ g) = −0.49, 95% CI −0.76 to −0.22; *p* < 0.001]. Between-study heterogeneity was low to moderate (τ^2^ = 0.03, I^2^ = 35.42%; Q = 4.69, *p* = 0.20).

The direction of effect remained consistent in the Knapp–Hartung sensitivity analysis (Supplementary Figure 2), with a wider confidence interval but retained statistical significance (SMD = −0.49, 95% CI −0.93 to −0.05; *p* = 0.04).

### 3.5 Vomiting outcomes

**FIGURE 4:**
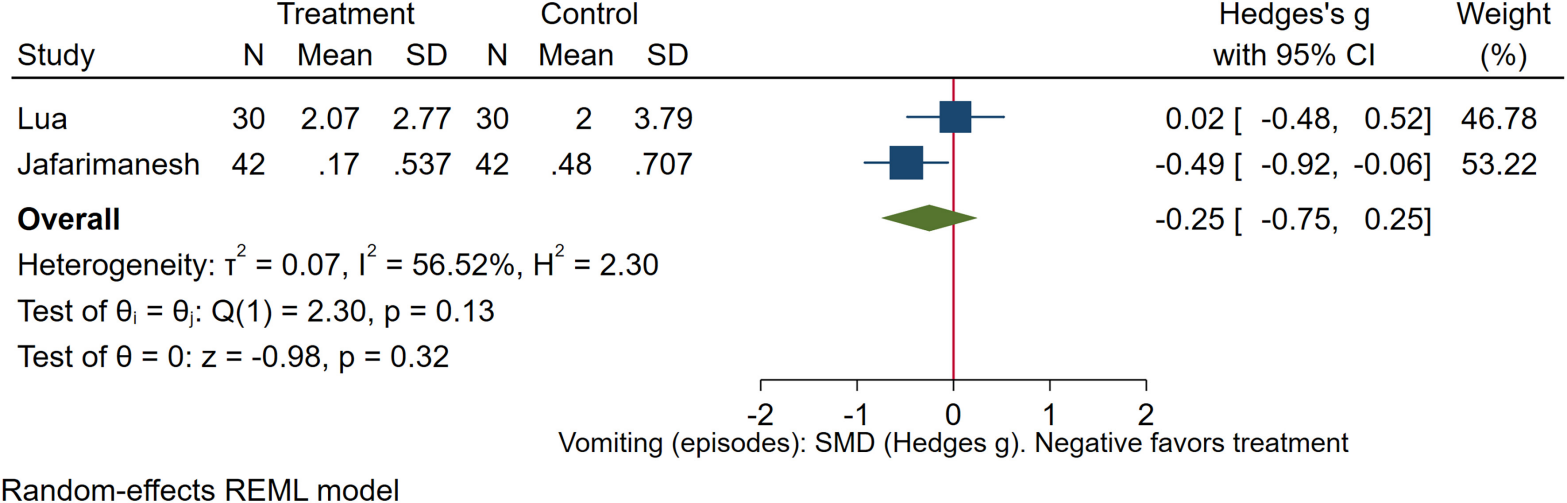
Forest plot of vomiting frequency (episodes) comparing essential oil–based interventions versus control. Effect sizes are presented as standardized mean differences [standardized mean differences (SMDs), Hedges’ g] with 95% confidence intervals using a random-effects restricted maximum likelihood (REML) model. Negative values favor essential oil–based interventions.

Two RCTs (*n* = 144) were included in the meta-analysis of vomiting frequency (episodes) (Figure 4). Essential oil–based interventions were not associated with a significant reduction in vomiting frequency compared with control [SMD (Hedges’ g) = −0.25, 95% CI −0.75 to 0.25; *p* = 0.32]. Between-study heterogeneity was moderate (τ^2^ = 0.07, I^2^ = 56.52%; Q = 2.30, *p* = 0.13). In the Knapp–Hartung sensitivity analysis (Supplementary Figure 3), the direction of effect was unchanged, but the confidence interval became substantially wider and remained non-significant (SMD = −0.25, 95% CI −3.48 to 2.98; *p* = 0.50).

**FIGURE 5:**
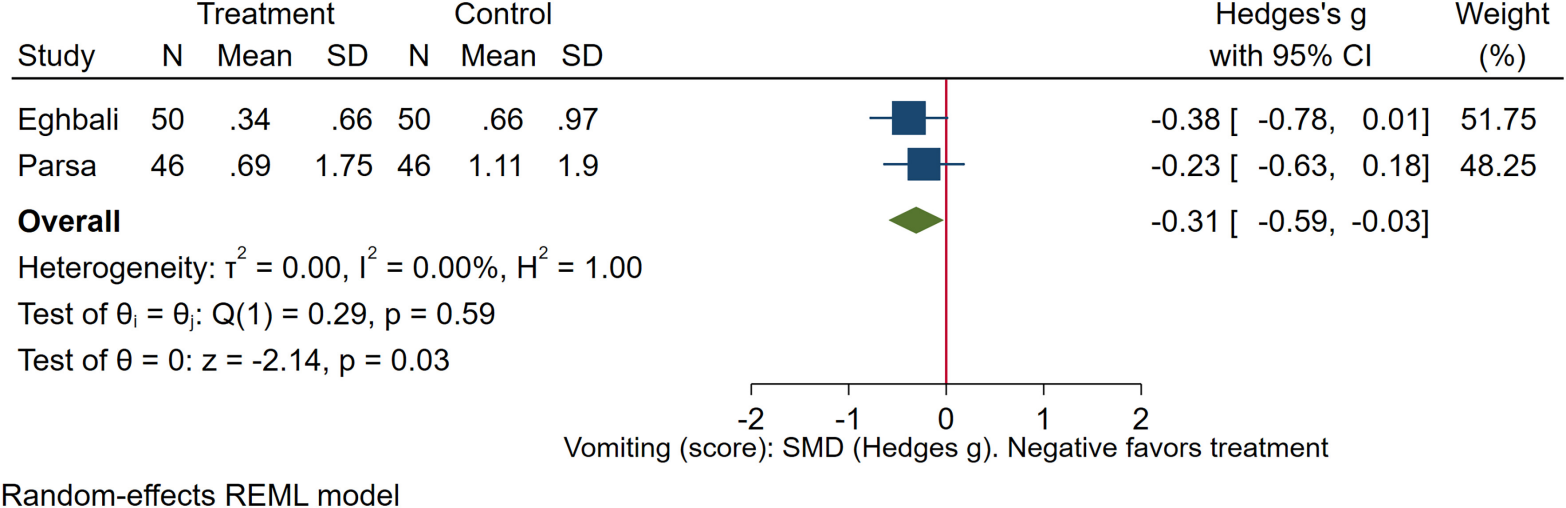
Forest plot of vomiting severity/score comparing essential oil–based interventions versus control. Effect sizes are presented as standardized mean differences [standardized mean differences (SMDs), Hedges’ g] with 95% confidence intervals using a random-effects restricted maximum likelihood (REML) model. Negative values favor essential oil–based interventions.

Two RCTs (*n* = 192) were included in the meta-analysis of vomiting severity/score (Figure 5). Compared with control, essential oil–based interventions were associated with a significant reduction in vomiting severity/score (SMD = −0.31, 95% CI −0.59 to −0.03; *p* = 0.03), with no observed heterogeneity (τ^2^ = 0.00, I^2^ = 0.00%; Q = 0.29, *p* = 0.59). In the Knapp–Hartung sensitivity analysis (Supplementary Figure 4), the pooled effect estimate remained in the same direction, but the confidence interval widened and statistical significance was no longer retained (SMD = −0.31, 95% CI −1.29 to 0.67; *p* = 0.16).

### 3.6 Additional analyses

Knapp–Hartung sensitivity analyses (Supplementary Figures 2–4) showed a consistent direction of effect across outcomes, with wider confidence intervals than the primary REML analyses. Statistical significance was retained for acute nausea severity, remained absent for vomiting frequency (episodes), and was no longer retained for vomiting severity/score after Knapp–Hartung adjustment. In the post hoc sensitivity analysis excluding the trial that used oral peppermint extract, the pooled effect for acute nausea severity remained statistically significant and in the same direction [3 RCTs, *n* = 252; SMD (Hedges’ g) = −0.38, 95% CI −0.63 to −0.14; *p* = 0.0024], with no observed heterogeneity (τ^2^ = 0.00, I^2^ = 0.00%). This finding suggests that the primary result for acute nausea severity was not solely driven by the oral peppermint extract trial. Formal assessment of publication bias (e.g., funnel plot asymmetry or Egger’s test) was not performed because the number of included studies per outcome was too small for reliable evaluation.

### 3.7 Adverse events and tolerability

Adverse events and tolerability were incompletely reported across the included trials, and the available data were insufficient for quantitative synthesis. No trial provided sufficient comparative safety data to pool intervention-related adverse events. The absence of systematic safety reporting should be considered a significant limitation of the current evidence base. This issue is particularly relevant because one included trial used oral peppermint extract rather than inhalation aromatherapy. Oral administration may have safety considerations that differ from inhalation-based interventions, including gastrointestinal discomfort, taste intolerance, reflux-related symptoms, or potential interactions with concomitant treatments. Therefore, although no quantitative safety signal could be estimated from the available studies, the safety and tolerability of essential oil–based interventions for CINV remain uncertain and should be prospectively and consistently assessed in future trials.

### 3.8 Certainty of evidence (GRADE)

The GRADE assessment is summarized in Table 2. The certainty of evidence was rated as low for acute nausea severity, mainly because of risk-of-bias concerns and possible publication or language bias related to the small evidence base and English-language restriction. The certainty of evidence was rated as very low for vomiting frequency and vomiting severity/score. For vomiting frequency, downgrading was mainly due to risk of bias, very serious imprecision from only two studies with a wide confidence interval crossing the null, and possible publication or language bias. For vomiting severity/score, downgrading was mainly due to risk of bias, serious imprecision from only two studies and loss of statistical significance after Knapp–Hartung adjustment, and possible publication or language bias. Overall, the certainty of evidence was limited, particularly for vomiting-related outcomes.

**TABLE 2:** Summary of findings (GRADE): essential oil–based interventions versus control for chemotherapy-induced nausea and vomiting in patients with breast cancer.

Outcome	Studies (*n*)	Participants (*n*)	Pooled effect (random-effects SMD, Hedges’ g; 95% CI)	Heterogeneity (I^2^)	Certainty (GRADE)	Reasons for downgrading
Acute nausea severity (acute phase)	Four RCTs	336	−0.49 (−0.76 to −0.22)	35.4%	Low ⊕⊕○○	Risk of bias due to blinding and reporting concerns (−1); possible publication/language bias due to English-language restriction, small evidence base, and inability to formally assess small-study effects (−1)
Vomiting episodes (frequency)	two RCTs	144	−0.25 (−0.75 to 0.25)	56.5%	Very low ⊕○○○	Risk of bias (−1); very serious imprecision due to only two studies, small sample size, and wide confidence interval crossing no effect (−2); possible publication/language bias (−1)
Vomiting score (severity)	Two RCTs	192	−0.31 (−0.59 to −0.03)	0%	Very low ⊕○○○	Risk of bias (−1); serious imprecision due to only two studies and loss of statistical significance after Knapp–Hartung adjustment (−1); possible publication/language bias (−1)

CI, confidence interval; CINV, chemotherapy-induced nausea and vomiting; GRADE, Grading of Recommendations Assessment, Development and Evaluation; RCT, randomized controlled trial; SMD, standardized mean difference. Effect estimates are presented as standardized mean differences (Hedges’ g) from random-effects models with 95% CIs. Negative SMD values favor essential oil–based interventions. I^2^ describes the proportion of total variability attributable to between-study heterogeneity. Certainty ratings were judged using the GRADE approach. Possible publication/language bias was considered because searches were restricted to English-language publications, the number of included studies was small, and formal assessment of small-study effects was not possible.

## 4 Discussion

In this systematic review and meta-analysis of randomized controlled trials, essential oil–based interventions were associated with reduced acute nausea severity in women with breast cancer receiving chemotherapy. However, this finding should be interpreted cautiously because only four RCTs were included and the interventions, comparators, chemotherapy regimens, outcome scales, and assessment time points varied across studies. By contrast, the pooled effect for vomiting frequency was not statistically significant. Vomiting severity/score showed a reduction in the primary REML analysis, but this finding was not retained after Knapp–Hartung adjustment, indicating substantial uncertainty for this outcome. Risk-of-bias assessments identified one study at high risk of bias, and the GRADE assessment indicated low certainty for acute nausea and very low certainty for vomiting-related outcomes. Taken together, these findings suggest that essential oil–based interventions may have a potential adjunctive role in acute nausea control, whereas evidence for vomiting-related outcomes remains imprecise and uncertain.

The pattern observed in this meta-analysis—clearer benefit for acute nausea than for vomiting-related outcomes—is biologically plausible and aligns with prior literature. Nausea is a multidimensional and highly subjective symptom influenced by sensory perception, affective response, anticipatory distress, and autonomic regulation. Inhaled aromas and other essential oil–based interventions may therefore affect nausea partly through olfactory, emotional, and symptom-perception pathways. By contrast, vomiting is a more stereotyped reflex response involving brainstem-mediated emetic pathways and is more directly targeted by pharmacological antiemetic therapy. This difference may explain why the apparent signal was stronger for nausea than for vomiting-related outcomes. However, this biological rationale remains hypothesis-generating, and the present evidence is insufficient to establish a definitive mechanism of action. In a recent meta-analysis, Zhang et al. similarly noted that the discrepant effects on nausea versus vomiting may reflect distinct pathophysiological mechanisms (8). This interpretation is also consistent with broader evidence suggesting that essential oil–based interventions may contribute through symptom perception, emotional regulation, and supportive care pathways, rather than replacing antiemetic pharmacotherapy (6).

Clinical heterogeneity was substantial despite low to moderate statistical heterogeneity for some outcomes. The included trials differed in essential oil type, route of administration, dose, exposure duration, chemotherapy regimen, background antiemetic protocol, comparator condition, symptom scale, and assessment time point. Interventions ranged from inhaled peppermint, ginger, or Citrus aurantium essential oils to an oral peppermint-derived preparation. Pooling was performed because all included studies addressed the same clinical question—whether essential oil–based supportive interventions are associated with acute CINV outcomes in women with breast cancer receiving chemotherapy—and because all extractable outcomes were continuous symptom measures that could be synthesized using standardized mean differences. A random-effects model was used to account for between-study variability. Nevertheless, this pooling should be interpreted as an exploratory broad-category synthesis rather than evidence for a single uniform intervention effect.

Outcome assessment also varied across studies. Nausea and vomiting were measured using different instruments and formats, including the Rhodes Index, VAS-based ratings, vomiting frequency, and vomiting severity/score. The use of SMD allowed statistical synthesis across different scales, but it does not fully eliminate conceptual and clinical differences between instruments. These differences may have influenced the magnitude and precision of the pooled estimates and further limit direct clinical interpretability, particularly for vomiting-related outcomes.

The oral peppermint extract trial requires specific consideration. This trial was retained under the broadened operational definition of essential oil–based interventions, but it should not be considered clinically equivalent to inhalation aromatherapy. Oral peppermint extract differs from inhaled essential oils in route of exposure, mechanism, systemic absorption, dosing, onset of action, and safety profile. In addition, because the original PROSPERO protocol was focused on aromatherapy and the eligibility definition was broadened after the initial screening process, the inclusion of the oral peppermint extract trial represents a protocol deviation/amendment. We therefore interpret the primary analysis as an exploratory synthesis of broader essential oil–based interventions rather than as evidence for inhalation aromatherapy alone. To examine whether the primary acute nausea finding was driven by the oral peppermint extract trial, we conducted a post hoc sensitivity analysis excluding this study. The pooled effect for acute nausea severity remained statistically significant and in the same direction. Nevertheless, this sensitivity analysis does not resolve the protocol-deviation issue or the clinical heterogeneity introduced by the oral route. The results should therefore be interpreted separately: the inhalation aromatherapy trials provide the more directly applicable evidence for aromatherapy practice, whereas the oral peppermint extract trial contributes exploratory evidence within a broadened essential oil–based intervention category.

These pooled estimates should be interpreted as an exploratory synthesis of essential oil–based supportive interventions as a broad category. They should not be interpreted as definitive evidence for any specific essential oil, dose, route, frequency, duration, or administration schedule. This distinction is particularly important in the acute phase, where symptom dynamics may change rapidly within the first 24 h after chemotherapy. Broader cancer-focused aromatherapy reviews and Cochrane-based evidence suggest possible short-term symptom-relief signals, but they also emphasize clinical heterogeneity, methodological limitations, and the need for better-standardized trials (6, 8, 22). Available related evidence outside the present synthesis also includes small pilot studies and safety reviews based largely on case reports or case series, which should be interpreted as contextual rather than definitive efficacy evidence (23, 24).

Our findings are broadly concordant with previous evidence syntheses, but they should be viewed as a focused and preliminary estimate for a clinically specific subgroup. Ahn et al. (6) reported an overall beneficial effect of aromatherapy on nausea/vomiting symptoms in patients with cancer, while also noting that studies evaluating vomiting alone often yielded non-significant results. This pattern is consistent with our findings. The signal was strongest for acute nausea, whereas vomiting frequency and vomiting severity were less consistent and became more uncertain after Knapp–Hartung adjustment. Previous evidence syntheses, including cancer-focused aromatherapy reviews and Cochrane-based evidence on massage with or without aromatherapy, have suggested possible short-term symptom relief but emphasized methodological limitations and the need for higher-quality trials (6, 8, 22).

The main added value of the present review is its narrow clinical focus. We restricted the analysis to women with breast cancer receiving chemotherapy and harmonized the primary synthesis to the acute phase within 24 h. This improves clinical interpretability compared with pan-cancer reviews. However, the small number of trials and the heterogeneity of intervention protocols limit the strength of inference. The current evidence supports only cautious consideration of essential oil–based interventions as adjunctive supportive care, particularly for acute nausea. These interventions should be integrated with, and not substituted for, guideline-based antiemetic management recommended in contemporary antiemetic guidelines (3).

Several limitations should be considered when interpreting these findings. First, the evidence base was very small, with only four randomized trials included overall and only two studies contributing to each vomiting-related outcome. This limited statistical power, reduced precision, and made the pooled estimates sensitive to study-level differences. Second, clinical heterogeneity was substantial. The included studies differed in essential oil type, administration route, dose, exposure duration, chemotherapy regimen, background antiemetic protocol, comparator condition, outcome scale, and assessment time point. Therefore, the pooled estimates should be interpreted as exploratory rather than as evidence of a uniform intervention effect. Third, one included trial used oral peppermint extract, which differs from inhalation-based aromatherapy in pharmacological exposure and safety considerations. Although the sensitivity analysis suggested that the acute nausea finding was not solely driven by this trial, the route-related heterogeneity remains clinically important. Fourth, the use of SMD for vomiting frequency may reduce clinical interpretability because vomiting episodes are count outcomes. This approach was used because the available aggregate data were reported as means and standard deviations, and individual participant-level count data were unavailable. Fifth, one included trial was judged to be at high risk of bias, and possible compromised blinding was a concern because nausea and vomiting were patient-reported or symptom-based outcomes.

Finally, formal assessment of publication bias was not performed because the number of studies per outcome was too small for reliable evaluation. Searches were limited to English-language publications and did not include clinical trial registries such as ClinicalTrials.gov or the WHO International Clinical Trials Registry Platform, nor did they include databases such as Scopus, CINAHL, or regional databases. Therefore, unpublished, ongoing, non-English, or database-specific studies may have been missed. This limitation may have contributed to publication or language bias and was considered when downgrading the certainty of evidence in the GRADE assessment.

Despite these limitations, the present findings suggest that essential oil–based interventions may be associated with a modest reduction in acute nausea severity in women with breast cancer receiving chemotherapy. However, this conclusion is based on a small, clinically heterogeneous, and low-certainty evidence base. The available evidence is insufficient to establish a definitive or clinically generalizable benefit. Evidence for vomiting-related outcomes remains inconsistent, imprecise, and very uncertain. Essential oil–based interventions should not be considered a substitute for guideline-based antiemetic therapy.

Future studies should focus on larger, multicenter randomized trials with standardized and clearly reported intervention protocols. These protocols should specify essential oil formulation, dose, route, frequency, timing relative to chemotherapy, and duration of exposure. Future trials should also standardize outcome definitions and assessment time points, with clear separation of acute and delayed CINV phases. More consistent reporting of chemotherapy emetogenic risk, background antiemetic regimens, adherence, adverse events, tolerability, and patient acceptability will be essential to improve comparability and strengthen the evidence base.

In conclusion, essential oil–based interventions may be associated with a modest reduction in acute nausea severity in women with breast cancer receiving chemotherapy. However, this finding should be interpreted cautiously because the evidence was limited to four RCTs with clinical heterogeneity and low certainty. Importantly, the evidence should not be interpreted as showing clinical equivalence between inhalation aromatherapy and oral peppermint extract. The inhalation aromatherapy trials provide the more directly applicable evidence for aromatherapy practice, whereas the oral peppermint extract trial contributes only exploratory evidence within a broadened essential oil–based intervention category. The inclusion of the oral peppermint extract trial also represents a protocol deviation/amendment from the original aromatherapy-focused PROSPERO registration and should be considered when interpreting the applicability of the findings. The current evidence is insufficient to establish a definitive clinically significant benefit or to support any specific essential oil, route, dose, or administration schedule. Evidence for vomiting-related outcomes remains inconsistent and very uncertain. These interventions should not replace guideline-based antiemetic therapy recommended in contemporary antiemetic guidelines (3). Well-designed, adequately powered multicenter randomized trials with prospectively registered protocols, standardized intervention definitions, consistent outcome measures, and systematic safety reporting are needed to clarify the role of inhalation aromatherapy and other essential oil–based supportive interventions for chemotherapy-induced nausea and vomiting.

## Data Availability

The original contributions presented in this study are included in this article/Supplementary material, further inquiries can be directed to the corresponding author.

## Supplementary material

Supplementary Figure 1 Summary of risk-of-bias judgments across included trials using the Cochrane RoB 2 tool. Weighted summary plot showing the proportion of studies judged as low risk, some concerns, or high risk for each RoB 2 domain and overall.

Supplementary Figure 2 Knapp–Hartung sensitivity analysis for acute nausea severity. Forest plot showing the pooled standardized mean difference (SMD, Hedges’ g) with 95% confidence interval for acute nausea severity using a random-effects REML model with Knapp–Hartung standard errors. Negative values favor essential oil–based interventions.

Supplementary Figure 3 Knapp–Hartung sensitivity analysis for vomiting frequency (episodes). Forest plot showing the pooled standardized mean difference (SMD, Hedges’ g) with 95% confidence interval for vomiting frequency using a random-effects REML model with Knapp–Hartung standard errors. Negative values favor essential oil–based interventions.

Supplementary Figure 4 Knapp–Hartung sensitivity analysis for vomiting severity/score. Forest plot showing the pooled standardized mean difference (SMD, Hedges’ g) with 95% confidence interval for vomiting severity/score using a random-effects REML model with Knapp–Hartung standard errors. Negative values favor essential oil–based interventions.
