# Supplementary material for: Essential oil–based interventions for chemotherapy-induced nausea and vomiting in women with breast cancer: a systematic review and meta-analysis
Source: Front Med (Lausanne). 2026 Jul 23;13:1897656. doi: 10.3389/fmed.2026.1897656 (PMC13441867; doi:10.3389/fmed.2026.1897656)
Supplement: Supplementary file 1 [file Table_1.docx]

Supplementary Material 1. Search strategies

**MEDLINE via PubMed**

Date searched: 14 February 2026

Limits: English-language publications

Records identified: n = 143

#1 Breast cancer

("Breast Neoplasms"[Mesh] OR breast cancer*[tiab] OR breast neoplasm*[tiab] OR breast tumor*[tiab] OR breast tumour*[tiab] OR breast carcinoma*[tiab] OR mammary cancer*[tiab] OR mammary neoplasm*[tiab] OR mammary carcinoma*[tiab])

#2 Chemotherapy

("Antineoplastic Agents"[Mesh] OR "Drug Therapy"[Subheading] OR chemotherap*[tiab] OR antineoplastic*[tiab] OR cytotoxic*[tiab])

#3 CINV / nausea / vomiting

("Nausea"[Mesh] OR "Vomiting"[Mesh] OR nausea[tiab] OR vomiting[tiab] OR emesis[tiab] OR retching[tiab] OR "chemotherapy-induced nausea and vomiting"[tiab] OR "chemotherapy induced nausea and vomiting"[tiab] OR CINV[tiab])

#4 Aromatherapy / essential oils

("Aromatherapy"[Mesh] OR aromatherap*[tiab] OR "aroma therapy"[tiab] OR "Essential Oils"[Mesh] OR "essential oil"[tiab] OR "essential oils"[tiab] OR "volatile oil"[tiab] OR "volatile oils"[tiab] OR "aromatic oil"[tiab] OR "aromatic oils"[tiab] OR inhalation[tiab] OR inhaled[tiab] OR olfactory[tiab] OR scent*[tiab] OR fragrance*[tiab] OR ginger[tiab] OR Zingiber[tiab] OR "Zingiber officinale"[tiab] OR peppermint[tiab] OR Mentha[tiab] OR "Mentha piperita"[tiab] OR "Citrus aurantium"[tiab] OR neroli[tiab] OR "orange blossom"[tiab] OR "bitter orange"[tiab])

#5 Study design filter

(randomized controlled trial[pt] OR controlled clinical trial[pt] OR randomized[tiab] OR randomised[tiab] OR randomly[tiab] OR placebo[tiab] OR trial[tiab])

Final combination

#1 AND #2 AND #3 AND #4 AND #5

**Embase**

Date searched: 14 February 2026

Limits: English-language publications

Records identified: n = 37

#1 Breast cancer

'breast cancer'/exp OR 'breast neoplasm*':ti,ab OR 'breast cancer*':ti,ab OR 'breast tumor*':ti,ab OR 'breast tumour*':ti,ab OR 'breast carcinoma*':ti,ab OR 'mammary cancer*':ti,ab OR 'mammary neoplasm*':ti,ab OR 'mammary carcinoma*':ti,ab

#2 Chemotherapy

'antineoplastic agent'/exp OR 'drug therapy'/exp OR chemotherap*:ti,ab OR antineoplastic*:ti,ab OR cytotoxic*:ti,ab

#3 CINV / nausea / vomiting

'nausea'/exp OR 'vomiting'/exp OR nausea:ti,ab OR vomiting:ti,ab OR emesis:ti,ab OR retching:ti,ab OR CINV:ti,ab OR 'chemotherapy-induced nausea and vomiting':ti,ab OR 'chemotherapy induced nausea and vomiting':ti,ab

#4 Aromatherapy / essential oils

'aromatherapy'/exp OR 'essential oil'/exp OR aromatherap*:ti,ab OR 'aroma therapy':ti,ab OR 'essential oil*':ti,ab OR 'volatile oil*':ti,ab OR 'aromatic oil*':ti,ab OR inhalation:ti,ab OR inhaled:ti,ab OR olfactory:ti,ab OR scent*:ti,ab OR fragrance*:ti,ab OR ginger:ti,ab OR Zingiber:ti,ab OR 'Zingiber officinale':ti,ab OR peppermint:ti,ab OR Mentha:ti,ab OR 'Mentha piperita':ti,ab OR 'Citrus aurantium':ti,ab OR neroli:ti,ab OR 'orange blossom':ti,ab OR 'bitter orange':ti,ab

#5 Study design filter

'randomized controlled trial'/exp OR 'controlled clinical trial'/exp OR randomized:ti,ab OR randomised:ti,ab OR randomly:ti,ab OR placebo:ti,ab OR trial:ti,ab

Final combination

#1 AND #2 AND #3 AND #4 AND #5

**Cochrane Central Register of Controlled Trials (CENTRAL)**

Date searched: 14 February 2026

Limits: English-language publications

Records identified: n = 32

("breast cancer" OR "breast neoplasm*" OR "breast tumor*" OR "breast tumour*" OR "breast carcinoma*" OR "mammary cancer*" OR "mammary neoplasm*" OR "mammary carcinoma*")

AND

(chemotherap* OR antineoplastic* OR cytotoxic*)

AND

(nausea OR vomiting OR emesis OR retching OR CINV OR "chemotherapy-induced nausea and vomiting" OR "chemotherapy induced nausea and vomiting")

AND

(aromatherap* OR "aroma therapy" OR "essential oil*" OR "volatile oil*" OR "aromatic oil*" OR inhalation OR inhaled OR olfactory OR scent* OR fragrance* OR ginger OR Zingiber OR "Zingiber officinale" OR peppermint OR Mentha OR "Mentha piperita" OR "Citrus aurantium" OR neroli OR "orange blossom" OR "bitter orange")

**Web of Science Core Collection**

Date searched: 14 February 2026

Timespan: Inception to 14 February 2026

Search field: Topic

Limits: English-language publications

Records identified: n = 23

TS=(

"breast cancer" OR "breast neoplasm*" OR "breast tumor*" OR "breast tumour*" OR "breast carcinoma*" OR "mammary cancer*" OR "mammary neoplasm*" OR "mammary carcinoma*"

)

AND

TS=(

"chemotherapy-induced nausea and vomiting" OR "chemotherapy induced nausea and vomiting" OR CINV

OR ((chemotherap* OR antineoplastic* OR cytotoxic*) NEAR/5 (nausea OR nause* OR vomiting OR vomit* OR emesis OR retching OR retch*))

)

AND

TS=(

aromatherap* OR "aroma therapy" OR "essential oil*" OR "volatile oil*" OR "aromatic oil*" OR inhalation OR inhaled OR olfactory OR scent* OR fragrance*

OR ginger OR Zingiber OR "Zingiber officinale"

OR peppermint OR Mentha OR "Mentha piperita"

OR "Citrus aurantium" OR neroli OR "orange blossom" OR "bitter orange"

)

**Additional searching**

Reference lists of included studies and relevant reviews were manually screened to identify additional eligible records. No additional records were identified through reference list screening.

Records identified from reference list screening: n = 0

Study selection notes

Records retrieved from all databases were imported into reference management software and deduplicated before title and abstract screening. Searches were conducted from inception to 14 February 2026 and were limited to English-language publications. Only randomized controlled trials were eligible for inclusion. Non-randomized, quasi-randomized, observational, review, and conference abstract-only reports without sufficient extractable data were excluded.
